# From benchmarking to best practices: Lessons from the laboratory quality improvement programme at the military teaching hospital in Cotonou, Benin

**DOI:** 10.4102/ajlm.v10i1.1057

**Published:** 2021-02-11

**Authors:** Alban Zohoun, Tatiana B. Agbodandé, Angélique Kpadé, Raliatou O. Goga, René Gainsi, Paul Balè, Bibata M. Sambo, Remi Charlebois, Rachel Crane, Michele Merkel, Ludovic Anani, Ekaterina Milgotina

**Affiliations:** 1 Department of Hematology, Faculty of Health Sciences, National University Hospital Center - Hubert Koutoukou Maga, Cotonou, Benin; 2 Army Teaching Hospital, University Hospital Center, Cotonou, Benin; 3 Global Scientific Solutions for Health (GSSHealth), Baltimore, Maryland, United States

**Keywords:** laboratory medicine, Stepwise Laboratory Improvement Process Towards Accreditation, SLIPTA, quality improvement, laboratory quality improvement, military laboratory, quality assurance, SLMTA, Strengthening Laboratory Management Toward Accreditation

## Abstract

**Background:**

In 2015, the Army Teaching Hospital–University Teaching Hospital (HIA-CHU [*Hôpital D’instruction des Armées de Cotonou Centre Hospitalier et Universitaire*]) laboratory in Benin launched a quality improvement programme in alignment with the World Health Organization Regional Office for Africa’s Stepwise Laboratory Improvement Process Towards Accreditation (SLIPTA). Among the sub-Saharan African laboratories that have used SLIPTA, few have been francophone countries, and fewer have belonged to a military health system. The purpose of this article was to outline the strategy, implementation, outcomes and military-specific challenges of the HIA-CHU laboratory quality improvement programme from 2015 to 2018.

**Intervention:**

The strategy for the quality improvement programme included: external baseline SLIPTA evaluation, creation of work plan based on SLIPTA results, execution of improvement projects guided by work plan, assurance of accountability via regular meetings, training of personnel to improve personnel competencies, development of external stakeholder relationships for sustainability and external follow-up post-SLIPTA evaluation.

**Lessons learnt:**

Over a period of 3 years, the HIA-CHU laboratory improved its SLIPTA score by 29% through a quality improvement process guided by work plan implementation, quality management system documentation, introduction of new proficiency testing and internal quality control programmes, and enhancement of personnel competencies in technical and quality management through training.

**Recommendations:**

The programme has yielded achievements, but consistent improvement efforts are necessary to address programme challenges and ensure continual increases in SLIPTA scores. Despite successes, military-specific challenges such as the high mobility of personnel have hindered programme progress. The authors recommend that further implementation research data be shared from programmes using SLIPTA in under-represented settings such as military health systems.

## Background

The Benin Army Health Service provides healthcare to army personnel, their families and civilians across Benin. The Benin Army Health Service is involved in disaster response and supports the Beninese public health system, with approximately 75% – 80% of its services provided to the civilian population. The Army Teaching Hospital–University Teaching Hospital (HIA-CHU [*Hôpital D’instruction des Armées de Cotonou Centre Hospitalier et Universitaire*]) of the Benin Army Health Service in Cotonou is a reference health centre known for the quality of its services and training. The HIA-CHU laboratory plays a leading role in the public healthcare continuum by providing diagnosis, screening, and initial and follow-up treatment as well as preventative care.^1^ Therefore, the HIA-CHU laboratory prioritises quality, in that it promotes the accuracy, reliability and timeliness of results.

In recent years, there has been an emphasis on improving medical laboratory services globally. Health priorities have been set forth by public declarations, such as the International Health Regulations and the Maputo Declaration (2008), in which signatories pledged to address and strengthen laboratory and health services.^2^ The International Standards Organization (ISO) 15198:2012 standard, ‘Medical Laboratories–Particular requirements for quality and competence’, serves as a standard for the laboratory quality management system (QMS) – a formalised system that outlines required structures and functions for medical laboratories. Progress made on the ISO 15189 standard can help a laboratory prepare for accreditation through a recognised agency; this allows the laboratory to demonstrate to clients, partners and staff that it has attained a high level of technical competence, thus instilling confidence in stakeholders on the accuracy and reliability of its results.

In sub-Saharan Africa, the clinical laboratory remains a weak link in the healthcare chain.^2,3^ Though progress has been made, most clinical laboratories in sub-Saharan Africa remain under-equipped, under-funded and far from attaining international norms and standards. Few laboratories are accredited to international quality standards, and most of these internationally accredited laboratories are based in South Africa.^4^ Quality assurance, which comprises QMS including the existence of a quality manual, use of internal quality controls (IQC) and participation in external quality assessment (EQA), is poorly implemented and often unavailable in many laboratories in sub-Saharan Africa.^5,6,7^ In the absence of quality assurance, there is the risk of laboratory errors, which could adversely impact patient care.

To alleviate these challenges and support awareness of the importance of achieving quality standards in medical laboratories in sub-Saharan Africa, the World Health Organization Regional Office for Africa developed in 2009 a phased laboratory quality management evaluation system called the Stepwise Laboratory Quality Improvement Process Toward Accreditation (SLIPTA).^8^ The African Society of Laboratory Medicine serves as the secretariat of the World Health Organization Regional Office for Africa SLIPTA programme. The SLIPTA framework guides improvement of performance, measures and evaluates the progress of laboratories towards ISO 15189 international accreditation and awards a certificate of recognition from zero to five-star ratings.^8,9^

The HIA-CHU in Cotonou, Benin, has a staff consisting of military and civilian personnel and includes a clinical pathologist, 12 medical laboratory scientists and support staff. The services provided include medical fitness evaluation, disease prevention, treatment and care, and teaching and research in medical, biological, pharmaceutical, paramedical, odontological and veterinary specialities. Laboratory services at HIA-CHU are versatile and the laboratory manages, on average, 20 000 patient files and 50 000 tests per year.

In January 2015, the HIA-CHU laboratory service initiated, for the first time, a quality improvement (QI) programme in alignment with the SLIPTA framework. The QI programme goal was to improve laboratory services related to disease diagnosis and monitoring via the implementation of QMS using SLIPTA framework. The QI programme aimed to provide training for laboratory personnel on quality management concepts and practices, monitor laboratory quality and adherence to quality systems, conduct laboratory test method validation and provide proficiency testing panels for HIV, tuberculosis and other critical tests – successful implementation of the QI programme ensures that laboratory stakeholders and end-users have confidence in laboratory data which informs clinical decisions and optimises patient care.

The HIA-CHU laboratory QI programme was launched through a collaboration with the US Department of Defense HIV/AIDS Prevention Program (DHAPP). As of 2015, the Benin Army Health Service already had a long-standing collaboration with DHAPP, which had been funding laboratory equipment and reagents, specifically to support HIV diagnosis and treatment monitoring. Over the years, both parties recognised that laboratory tests and equipment alone are not sufficient to demonstrate test result quality; tests and equipment must be accompanied by laboratory quality practices implemented by properly trained and motivated personnel. To address this recognised need, DHAPP began supporting the Benin Army Health Service in the execution of the HIA-CHU laboratory QI programme in 2015, with implementation assistance provided by the United States-based global health company Global Scientific Solutions for Health (GSSHealth).

The purpose of this article is to report on the implementation of the QI programme at the HIA-CHU laboratory from 2015 to 2018.

## Description of the Intervention

### Baseline SLIPTA evaluation and work plan creation

The laboratory QI programme began in January 2015 with initial programme planning among stakeholders (HIA-CHU leadership, DHAPP, GSSHealth) and the facilitation of a comprehensive baseline evaluation of the HIA-CHU laboratory by GSSHealth using the World Health Organization Regional Office for Africa SLIPTA checklist, version 2:2015. The baseline SLIPTA evaluation allowed the assessment of the 12 quality management sections: organisation and personnel, management reviews, process control and internal quality assessment and EQA, information management, corrective action, equipment, purchasing and inventory, documents and records, occurrence management and process improvement, internal audit, client management and customer service, and facilities and safety.

Following the evaluation, leaders from HIA-CHU, DHAPP and GSSHealth convened for a collaborative session to review findings, identify priority QI areas and develop a tailored QI approach for each priority area.

A tailored QI approach was developed to strengthen HIV testing processes – the focus of the funding agency DHAPP; it covered the identification of key quality indicators to track and improve upon over time and the development and execution of the QI work plan.

The key indicators identified to measure laboratory improvement based on ease of collection and relevance to the work plan included:

- SLIPTA: Percentage improvement in total and by section.
- EQA: Percentage score in PT programme.
- IQC: Accuracy of quality control results.
- Personnel training: Percentage change in theoretical test scores for training workshops.
- Laboratory documentation: Number of documents created and adequately implemented to standardise laboratory practices and formalise the commitment to the QMS.

The laboratory-set targets were: continual improvement in the SLIPTA score through the establishment and implementation of improvement projects tracked by work plans, participation in EQA and PT and improvement of performance where the score is less than 100%, participation in IQC activities and improvement of processes in case of discrepancies between expected and obtained results, strengthening of laboratory quality management processes guided by well defined documentation, and the advancement of laboratory personnel competencies through training and mentorship workshops.

**FIGURE 1:**
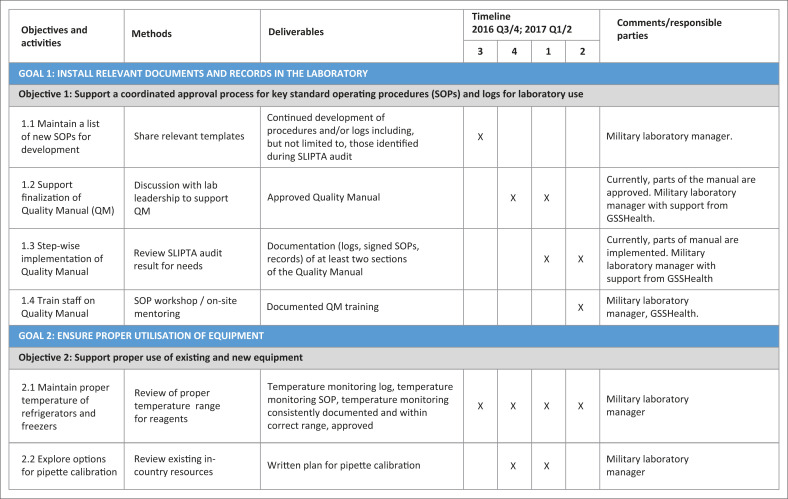
Example of a quality improvement work plan (first page) developed by the Army Teaching Hospital–University Teaching Hospital laboratory, Benin, 2016–2017.

The HIA-CHU laboratory supervisor facilitated work plan development in collaboration with partners (DHAPP and GSSHealth) and laboratory staff. The work plan included high-level QI goals, specific objectives for each goal and details of specific, measurable, achievable, realistic, and timely, or ‘SMART’, tasks and deliverables to meet set targets within defined timeframes (see Figure 1).

The work plan was progressively implemented and updated through improvement project execution, progress monitoring, ongoing data collection and review, and continual alignment with the funder’s priorities. Staff were engaged in the work plan implementation through their appointments to specific QI projects with oversight by the laboratory supervisor, and through continuous engagement in recurring collaborative work plan update meetings to promote accountability. Quality improvement activities conducted in the context of the work plan included: nomination of new QMS personnel roles (quality manager, biosafety manager), targeted laboratory personnel training and mentorship, implementation of EQA and IQC, definition of essential QMS processes and development and use of associated documentation, and coordination of a follow-up SLIPTA evaluation to measure progress.

## Lessons learnt

From the baseline SLIPTA evaluation, the identified priority issues, which correspond to low-scoring SLIPTA sections, include the following: the lack of a quality manual or safety manual, the lack of standard operating procedures for all processes and technical procedures, the lack of PT and quality control for tests, the lack of equipment maintenance and repair logs, the lack of temperature monitoring processes, and the lack of mistakes or error logs.

The initiation and implementation of a QI programme at the HIA-CHU laboratory yielded numerous positive changes as perceived by HIA-CHU hospital leadership, laboratory management and staff, and partners (DHAPP and GSSHealth). Positive changes included the establishment of a laboratory quality management team, achievement of designated QI projects resulting in SLIPTA score improvements, the improvement of IQC and EQA test result accuracy, the development and implementation of over 50 standard operating procedures, and the strengthening of personnel competencies through targeted QMS training and mentorship. Throughout the years of the QI programme, support from funding partner DHAPP and implementing partner GSSHealth ensured continuous evaluation of processes via the internal and external evaluations and the update of work plans and associated QI projects.

### Staff training and work plan updating

The engagement of laboratory staff was key to the establishment and implementation of the laboratory QI work plan at HIA-CHU. To encourage staff commitment to the QI approach and ensure staff in-depth orientation to quality management concepts and testing processes, the laboratory QI approach prioritised staff training and mentorship.

**FIGURE 2:**
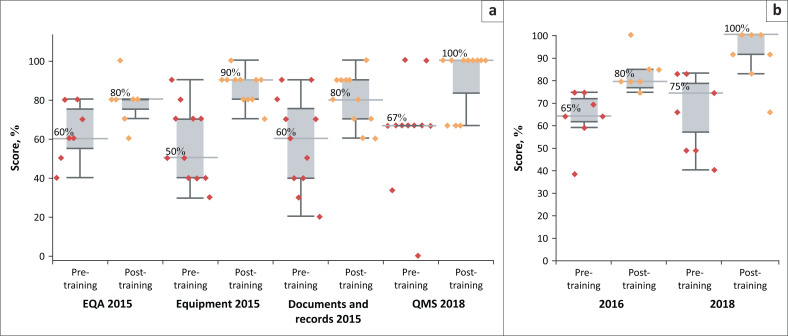
Pre- and post-training workshop theoretical test scores of military laboratory staff from quality management (left panel) and biosafety (right panel) workshops, Benin, 2015–2018. Box plots illustrate the distribution of scores among trainees (red diamonds for pre-training and yellow diamonds for post-training) and grey horizontal lines with data labels indicate median score across all the trainees. The interquartile range shows how the data are dispersed by dividing the data into quartiles (depicted by the dark and grey horizontal lines). (a) QMS training: test scores, (b) BS&S training: test scores.

Over 50 technicians participated in training workshops on QMS co-organised and co-facilitated by the Benin military health system and GSSHealth. All training sessions consisted of didactic and interactive sessions and tests were administered pre- and post-training to evaluate knowledge change among the trainees. The median change in test score from pre-training to post-training for all the trainees increased from 15% to 40% (Figure 2). Improvements in participants’ theory test results demonstrated improved understanding of QMS and technical concepts and facilitated personnel participation in QI projects.

In the first year of the programme, two quality workshops were held for laboratory staff with GSSHealth facilitators. The objectives of the workshops were to improve the competency of laboratory staff in the areas of quality assurance, QMS and HIV and tuberculosis testing. These topic areas were priorities for the laboratory director and DHAPP – the funder, whose focus is the prevention and control of HIV and HIV comorbidities. The first workshop took place in April 2015 with a focus on quality management concepts for equipment management, document writing and EQA. Staff received guidance and mentorship on HIV rapid testing. A second workshop took place in September 2015, with a focus to increase staff competency on laboratory supply chain and stock management. At the end of the first year, a follow-up SLIPTA evaluation was conducted.

In 2016, the QI programme continued with an updated work plan, and laboratory staff participated in a national biosafety and biosecurity workshop hosted by the Ministry of Defense and the Ministry of Health. The second year concluded with a poster presentation by the HIA-CHU laboratory supervisor on the quality programme at the African Society of Laboratory Medicine international conference in December 2016.

In 2017, the laboratory quality team updated their quality work plan, worked with laboratory personnel on QI projects, implemented the use of quality controls for CD4 testing, and organised an internal SLIPTA evaluation. In April 2017, one laboratory staff member from HIA-CHU was sponsored to attend a multi-country, 5-day training workshop in Senegal. The workshop covered essential aspects of laboratory quality management, including document management and standard operating procedure writing; equipment management and maintenance; error occurrence management, prevention and corrective action, and quality indicators. Finally, a follow-up external SLIPTA evaluation was conducted in October 2017 to re-evaluate the laboratory system, measure progress since the previous external SLIPTA evaluation, and update the QI programme.

As the QI programme moved into its third year in 2018, the laboratory again updated its work plan, based on the previous evaluation, and revised and updated key documentation including the laboratory quality manual and biosafety manual. In April 2018, a joint Ministry of Defense and Ministry of Health laboratory biosafety and quality management workshop was organised, to increase staff competency on principles and ISO standards for biosafety and biosecurity.

Through its ongoing commitment to training personnel in both technical and quality management topics, the laboratory has observed significant improvements in staff competencies and performance. For example, the SLIPTA sections in which the laboratory achieved the most significant quality improvements correspond to the quality management topics covered during training workshops. Data from the QI programme showed that post-training, personnel knowledge was improved and retained (Figure 2). Additionally, in 2015, staff undertook training on HIV rapid testing processes; thereafter, their HIV PT scores reached 100%.

### Document creation and implementation

The laboratory document management system has been developed gradually over time and has included the creation and implementation of a quality manual, a biosafety manual, a sample collection manual and more than 50 standard operating procedures, technical instructions, forms and logs (e.g. temperature monitoring logs, corrective and preventive action forms). The laboratory supervisor and designated quality manager took the lead in developing documents, executing a document management process, and introducing new documents into circulation in the laboratory. Laboratory personnel were oriented to new documents and procedures during regular laboratory meetings, facilitating the understanding and proper use of new documents. The availability of procedures and other documents helped laboratory management ensure that personnel observed standardised processes across the pre-analytical, analytical and post-analytical phases, and simplified the training of new personnel. Furthermore, to ensure that only correct and updated documents were available in the laboratory, processes to control document revision, approval and version release were instituted by the laboratory supervisor and quality team.

### External quality assessment and internal quality control results

In September 2015, the HIA-CHU laboratory enrolled and participated in an annual PT programme for HIV serology with commercial EQA provider Thistle quality assurance, and later with the Benin Ministry of Health, to promote sustainability. During the four EQA events for HIV serology, nine technicians participated, and PT scores of 100% were obtained. No corrective action was needed to improve proficiency; laboratory personnel were nonetheless oriented on root-cause analysis, and corrective and preventive action in case of future instances of lower EQA scores.

In 2018, the HIA-CHU laboratory participated in two commercial haematology EQA programmes with Human Quality Assessment Services. The laboratory results for the first haematology EQA test were in the acceptable range for all analytes except for monocytes or mid cells, due to a reporting error. As a corrective action, the laboratory established a system for the supervised recording of results, in which the laboratory section supervisor verifies the accuracy of test results entered manually by technicians, after which results are entered into a computer by a secretary and printed for final validation. This process has helped reduce transcription errors.

The HIA-CHU also began an IQC programme in biochemistry for the first time, in which the laboratory used high, normal and low commercial controls on equipment daily. When control values are outside the acceptable range, the laboratory staff will conduct a root-cause investigation and analysis. Elements to be investigated include reagents (conservation, expiration, etc.), room temperature, quality of distilled water, quality of the control product, etc. If the issue persists after the above steps, the equipment is then recalibrated. All corrective actions and measures taken are documented and recorded.

### Improvement of SLIPTA score

**FIGURE 3:**
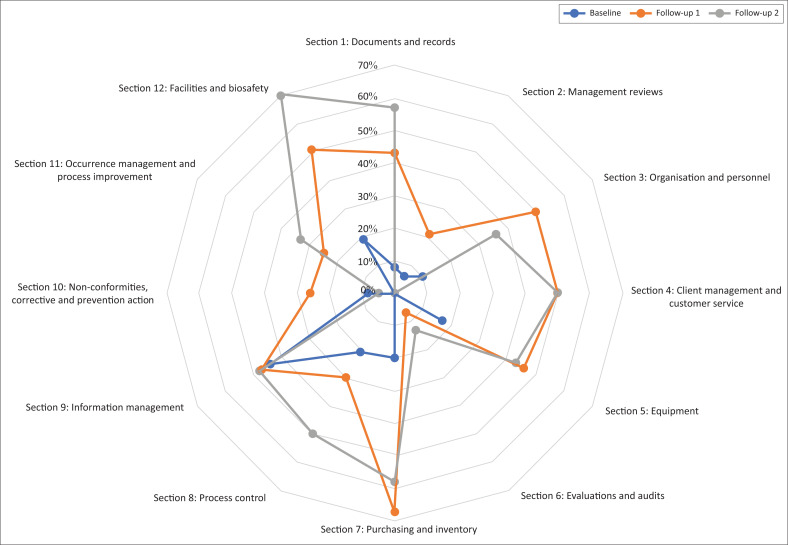
Performance of the Army Teaching Hospital–University Teaching Hospital laboratory in each section of the Stepwise Laboratory Improvement Process Towards Accreditation checklist, Benin, 2016-2018. Baseline evaluation, September 2015; Follow-up evaluation 1, January 2016; Follow-up evaluation 2, October 2017.

Overall improvement of the laboratory’s quality system was demonstrated by a 29% increase in SLIPTA score, from 16% at baseline evaluation in 2015 to 45% at the follow-up evaluation in 2017 (see Figure 3).

Since 2018, reliance on the Ministry of Health National HIV Reference Laboratory for the administration of an HIV serology PT programme has allowed the HIA-CHU laboratory and other military laboratories to ensure a more sustainable and affordable PT option.

Several important factors can explain the successes achieved during the QI programme. The laboratory leadership and staff benefited from the strong support of the hospital management, thus underpinning laboratory staff motivation to sustain quality improvements. Also, the implementing partner provided support in the execution of work plans, drafting of procedures, funding of training visits and organisation of regular teleconferences to track progress. The implementing partner provided sound advice for the appropriation of the quality approach and the implementation of improvement steps.

## Recommendations

Although SLIPTA is an adaptable structure and was used to guide the HIA-CHU laboratory’s quality successes, its formal implementation in francophone African countries and military contexts has been limited.^8^ Military health systems face unique challenges that impact their laboratory QI efforts; the challenges and associated responses of HIA-CHU laboratory may be relevant to other military laboratory programmes or to non-military laboratories that have limited funds to invest in QI efforts. Regardless of improvements made, QI is a never-ending process and the sustainability of gains is not guaranteed.^10^ Despite the quality advancements made over the past several years, challenges remain, requiring corrective action to ensure the efficacy of the ongoing programme.^11,12,13^ The issue of the high mobility of military personnel has made it difficult to fully and consistently integrate staff into quality laboratory operations and maintain the laboratory QMS. As a result of high staff turnover – a key facet of the military system – all members of the starting laboratory quality team are no longer in service at the HIA-CHU laboratory in Cotonou. Continuous staff turnover in part explains the slowdown in progress observed between the 2016 and 2017 follow-up evaluations. Staff mobility has also negatively impacted the timelines for the execution of planned activities such as management reviews, internal evaluations and inventory processes, for all of which personnel must be properly trained and oriented to ensure their execution per SLIPTA and ISO 15189 requirements.

**Table:** 

Lesson learned
The implementation of a QI approach supported by improved documentation and record-keeping has increased staff and end-user confidence in the reliability and reproducibility of laboratory test results.
Support from the hospital hierarchy is essential to ensure the necessary time and resources are allocated to quality improvement processes.
For a QI initiative to be effective and sustainable, all laboratory personnel should be engaged in the process, with quality management roles well defined.
Staff mobility in military settings renders the ongoing implementation of a QI programme more challenging; military sites should take into account the need to engage all laboratory personnel in a QI mindset and should plan backup QI roles in case of personnel deployment.

Although the laboratory QI programme has incorporated training workshops, the programme would benefit from the establishment of a structured framework for continous personnel training allowing the laboratory to maintain a pool of trained and dedicated personnel.

Going forward, the ambition of HIA-CHU laboratory is to continue advancing in laboratory quality using the SLIPTA guideline, to ultimately achieve a five-star SLIPTA rating and become internationally accredited to the ISO 15189 standard. The HIA-CHU laboratory also has the support of the Benin military health system authorities to expand the QI programme to multiple laboratory sites, prioritise the accreditation of clinical laboratories, and to integrate accreditation programmes into health sector policy and development programmes. To date, the HIA-CHU laboratory has expanded the QI programme to military clinical laboratories in Ouidah, Porto-Novo and Parakou. Military laboratory leaders envision the further expansion of the QI programme to additional military sites using a network approach.
